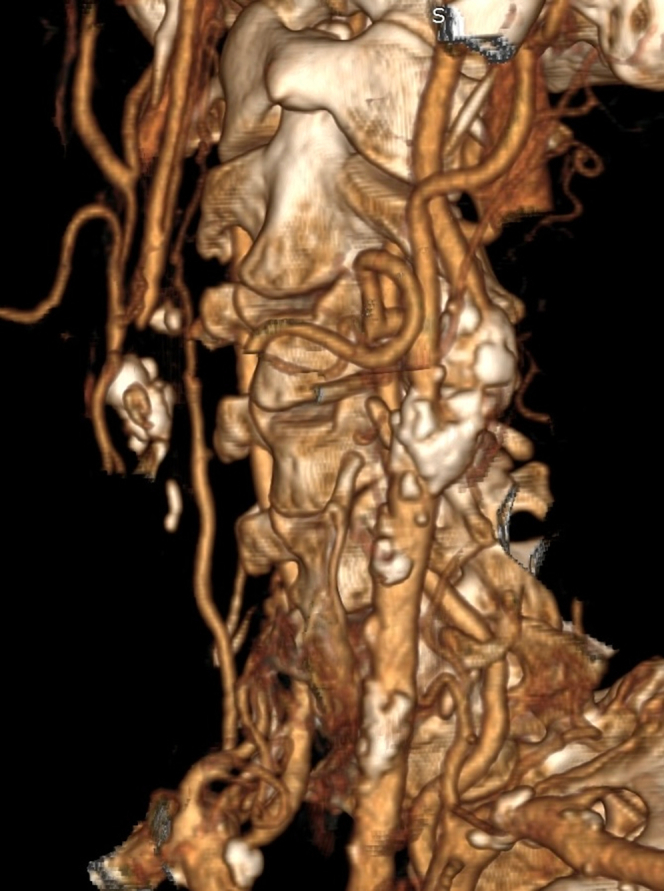# Subclavian–carotid bypass for occluded common, internal, and external carotid arteries with anomalous ascending pharyngeal collateral maintaining internal carotid flow

**DOI:** 10.1016/j.jvscit.2026.102306

**Published:** 2026-05-08

**Authors:** Grigol Keshelava, Zurab Robakidze, Devi Tsiklauri

**Affiliations:** Department of Vascular Surgery, Clinic Healthycore, Tbilisi, Georgia

A 61-year-old man with hypertension, hyperlipidemia, and tobacco use presented with four episodes of transient dysarthria over 2 months without optimized medical therapy. Ultrasonography and computed tomography angiography revealed right common carotid artery, proximal internal carotid artery (ICA), and external carotid artery occlusion with distal ICA patency maintained by retrograde flow via an anomalous ascending pharyngeal artery (APA) originating from its middle segment. Additional findings included >90% left ICA stenosis and >90% right vertebral artery stenosis (*A*/Cover*, B*). No infarcts were seen on magnetic resonance imaging and computed tomography.

The patient provided written informed consent for publication.

Given the patient’s right-hand dominance and left hemispheric symptoms, left carotid endarterectomy was performed first under general anesthesia and shunting, completed with patch angioplasty. The patient was discharged on day 3 in good condition with dual antiplatelet therapy and statin.

One month later, right-sided reconstruction was performed because only limited collateral flow from the APA was considered insufficient for long-term cerebral perfusion stability, aggravated by vertebral artery stenosis. The procedure involved supraclavicular exposure of the subclavian artery for end-to-side anastomosis to a ringed polytetrafluoroethylene prosthesis. Cervicotomy exposed the bifurcation. The ICA and external carotid artery were transected distal to the occlusions (ICA was clamped proximal to the APA to preserve collateral flow). A new carotid bifurcation was created and followed by the end-to-end anastomosis to the prosthesis (*C*). Ringed polytetrafluoroethylene was chosen for its kink resistance, durability, and excellent long-term patency.

Flow was restored without complications. The patient was discharged on day 4 with dual antiplatelet therapy.

Anomalous origin of the APA from the ICA is rare (0.14%-6%). This variant preserved patency amid occlusion, facilitating bypass. Similar cases describe an APA maintaining flow in occluded ICAs treated by stenting or endarterectomy.^1^^,^^2^

**Figure:**
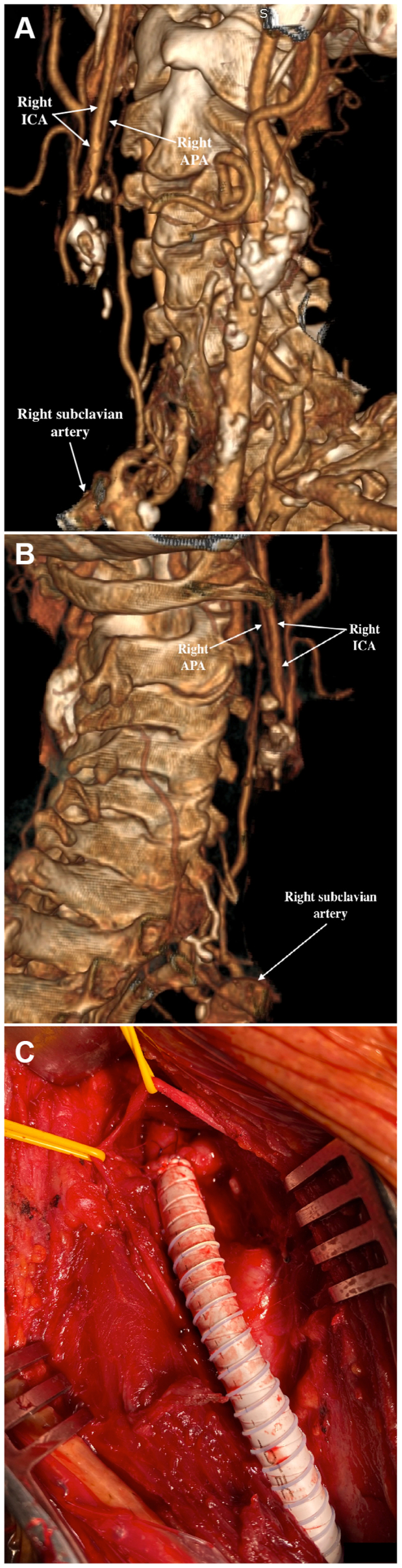

## Funding

None.

## Disclosures

None.

## Figures and Tables

**Cover image:**